# A cost-effective modular laboratory solution for industrial automation and applied engineering education

**DOI:** 10.1016/j.mex.2025.103388

**Published:** 2025-05-28

**Authors:** Musa Al-Yaman, Dana Alswaiti, Adham Alsharkawi, Majid Al-Taee

**Affiliations:** a Mechatronics Engineering Department, University of Jordan, Amman, Jordan; b Independent Consultant of Computing and Systems Engineering, Liverpool, UK

**Keywords:** Applied engineering education, Interactive training experience, Hands-on learning, Modular lab design, Programmable logic controller, Simulation kits, *Modular and Transferable PLC Method*

## Abstract

In today’s digital landscape, affordable Programmable Logic Controllers (PLCs) for industrial automation and applied engineering education are essential, as traditional setups are often too costly for many institutions and individuals. In this paper, a cost-effective, modular PLC lab was designed and developed to enable hands-on learning in industrial automation. The lab accommodates ten pairs of trainees, allowing for simultaneous experimentation using modular simulation kits tailored for various applications, such as traffic light systems, elevators, and automated filling systems. Trainees can prepare, test, and verify their code on these kits in real-time, enhancing their hands-on experience with programmable logic controllers. Each kit offers a set of features, including an integrated input/output interface that supports a variety of input types (e.g., switches, sensors, and push buttons) and output options (e.g., lights, motors, and actuators). This well-rounded setup is specifically designed to meet the educational and training objectives of the lab, promoting an interactive learning environment that develops essential automation skills and builds trainee confidence in applying PLC technology across real-world scenarios.

• Offers a cost-effective PLC solution for industrial automation and engineering education, addressing the high cost of traditional systems.
• Provides a scalable PLC lab setup developed to facilitate hands-on learning and practical experience in industrial automation, enabling simultaneous experimentation and collaborative learning.
• Aligns with educational objectives, fostering an interactive, hands-on environment that builds essential automation skills, by enabling real-code preparation, testing and verification.

## Graphical abstract

**Figure:**
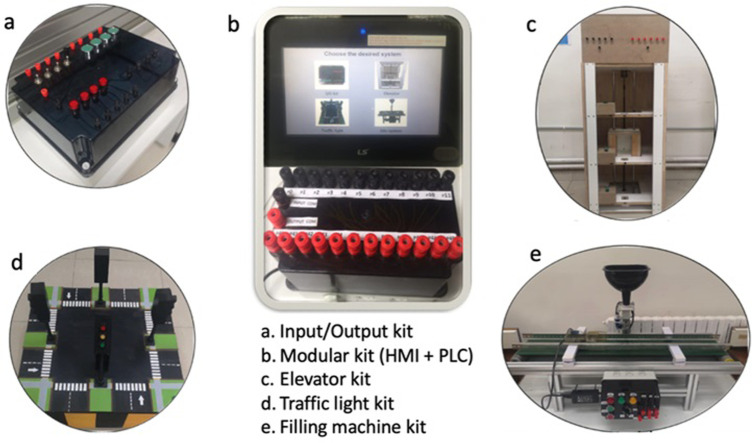

Specifications table

**Table:** 

Subject area:	Engineering
More specific subject area:	*Mechatronics Engineering*
Name of your method:	*Modular and Transferable PLC Method*
Name and reference of original method:	*Not applicable*
Resource availability:	*Software:* *PLC software: XG5000 (Vendor: LS Electric)* *HMI software: XP-Builder (Vendor: LS Electric)* *Hardware:* *PLC: XEC-RD32H (Vendor: LS Electric)* *HMI: eXP60-TTA (Vendor: LS Electric)*

## Background

In today's world, addressing various challenges, particularly in lower-middle-income countries, often involves considerations of cost-effectiveness and resource optimization [1]. This effort is driven by making technology and resources more accessible and affordable. For instance, low-cost automated devices are increasingly being utilized in laboratories, as demonstrated by practical implementations such as automation training devices [2] and affordable laboratory equipment [3]. Open-source tools like Arduino-Simulink interfaces have facilitated the development of cost-effective feedback control systems [4]. In parallel, advancements in education have introduced low-cost robotic platforms for hands-on learning [5] and modular training kits aimed at industrial automation [6]. Similarly, advancements in smart PLC systems are enabling cost-effective solutions in Industry 4.0 production environments [7]. Portable industrial automation training kits have also been designed to support modular education [8], and programmable logic controller training platforms are being used for process control applications [9].

In 2015, Kuniaki Yajima et al. [10] developed a learning kit known as the “Sequence Control Kit,” which included a set of sensors and a PLC. The kit was designed to move a box to the position of a ball, as detected by one of the sensors. In this learning kit, a teacher can adjust the difficulty level by modifying the complexity of the tasks or configurations. Additionally, students can learn the fundamental functionality of a PLC and how to use and configure it effectively. In 2017, Sukir et al [11] developed a conveyor trainer kit integrated with a monitoring system and PLC module, providing enhancements over conventional training kits. Later, in 2019, they introduced a PLC-based electrical machine trainer kit designed to support a variety of practical learning activities [12].

Building on advancements in industrial automation, Burhan et al. [13] introduced a PLC kit designed to enhance students' practical skills by offering hands-on experience with real-world components such as I/O modules, DC motors, and relays. Maarif et al. [14] has also designed a portable “industrial automation kit” that simplifies PLC usage through modular components and portability. It includes a controller module, plug-and-play I/O interface, DC motor module, and electro-pneumatic module which are standard parts in the industry field. It is easy to deal with outside the framework of laboratories. Ahmad Khairudin et al. [8] designed a small, light, and portable ‘Industrial Automation Education Training Kit’ to bridge gaps such as limited access to industrial tools and lack of practical skills in academia.

Numerous researchers have developed cost-effective industrial training kits. For example, Akparibo et al. [9] introduced a portable PLC training platform for industrial process control, priced at $214 in 2016. Although the exact costs of other kits are not always disclosed, similar platforms are generally estimated to range between $200 and $500, depending on the components used and system complexity. Castillo [15] designed an Electro-Pneumatic Automation Trainer that adheres to industry standards and offers ease of use, demonstrating high efficiency. Similarly, Kheiralla [16] developed a PLC workbench that includes essential laboratory modules such as latching, timer, and counter functions.

In contrast to these more limited solutions, this paper presents the design and experimental implementation of a comprehensive automation lab setup. A full automation lab solution is defined here as one that supports a wide range of applications, allows multiple trainees to work concurrently, and offers an extensive learning experience across various facets of industrial automation. Unlike most existing works that focus on one or two standalone kits intended for partial lab integration, the proposed solution addresses the broader needs of scalable, multi-user training environments. This work focuses on the hardware aspects of the lab while the software part is considered beyond the scope of this paper and will be the subject of a future publication. The proposed lab can accommodate ten pairs of trainees, allowing for simultaneous experimentation using modular simulation kits tailored for various applications, such as traffic light systems, elevators, automated filling systems, and others. However, for conciseness, the various aspects of the lab are presented with a particular reference to a pair of sample application kits: input/output systems, traffic light systems, elevators, and automated filling systems. Trainees can utilize these kits to prepare, test, and verify their code in real-time, significantly enhancing their hands-on experience with programmable logic controllers (PLCs).

By engaging directly with practical applications, learners develop a deeper understanding of automation principles and the intricacies of industrial control systems. These training kits are designed to simulate real-world scenarios, providing a dynamic platform for troubleshooting, iterative coding, and system optimization Each kit is equipped with a comprehensive set of features that cater to diverse learning and operational needs. Central to their functionality is an integrated Input/Output (I/O) interface, which supports a wide range of input types, including switches, sensors, and push buttons. These inputs allow trainees to explore various data acquisition methods and control logic pathways.

On the output side, the kits provide versatile options such as indicator lights, motors, actuators, and solenoid valves. This diversity enables learners to interact with typical industrial components, enhancing their ability to design, implement, and test automation systems effectively. Furthermore, these kits often incorporate plug-and-play modules, making it easy to set up and modify configurations without requiring specialized tools or extensive technical expertise. Many include built-in monitoring and diagnostic features, allowing trainees to observe system behaviour in real-time and refine their solutions for improved performance. Such hands-on experience helps bridge the gap between theoretical knowledge and practical application, fostering a more comprehensive understanding of PLC operations and their role in industrial automation.

## Method details

**Fig. 1:**
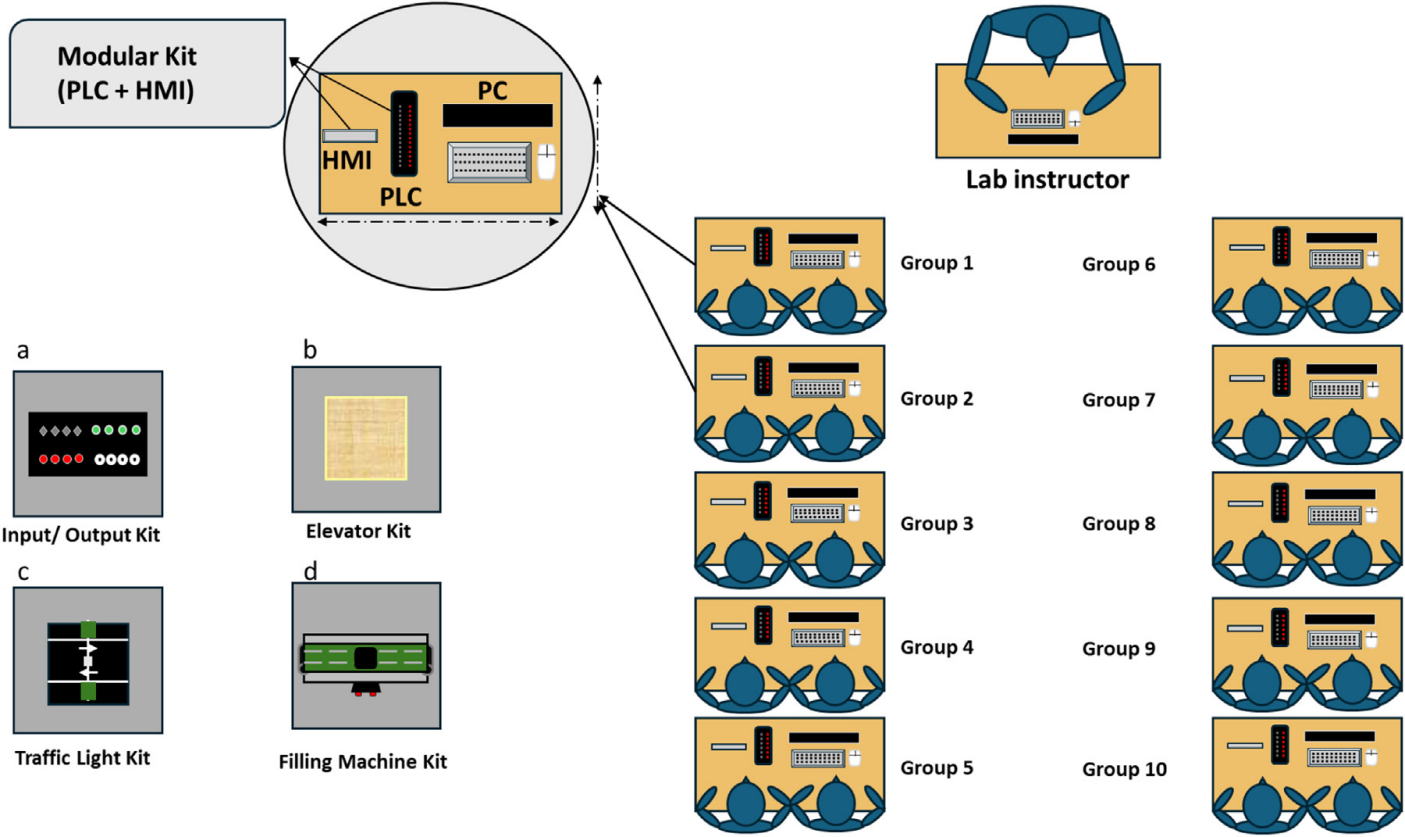
The proposed lab layout with learners and kit distributions. a. input/output; b. elevator; c. traffic light; d. filling machine.

The layout of the proposed laboratory, including learner and kit distributions, is depicted in Fig. 1. The lab is designed to accommodate a maximum of 20 students, organized into ten groups of two students each. This arrangement ensures that all groups can conduct the same experiment simultaneously, fostering a collaborative and interactive learning environment. The lab setup features two primary categories of hardware components:

(1) *Modular Kit*: Ten Modular Kits, each equipped with a PLC and a Human-Machine Interface (HMI). Each group is assigned one Modular Kit, providing them with the necessary tools to independently perform experiments and develop practical skills.
(2) *Application Kits*: A selection of specialized kits simulating real-world industrial scenarios. These kits include I/O interface, a traffic light control kit, an elevator kit, a filling machine kit, and potentially other applications. To optimize costs, one application kit of each type is shared among all groups.

**Fig. 2:**
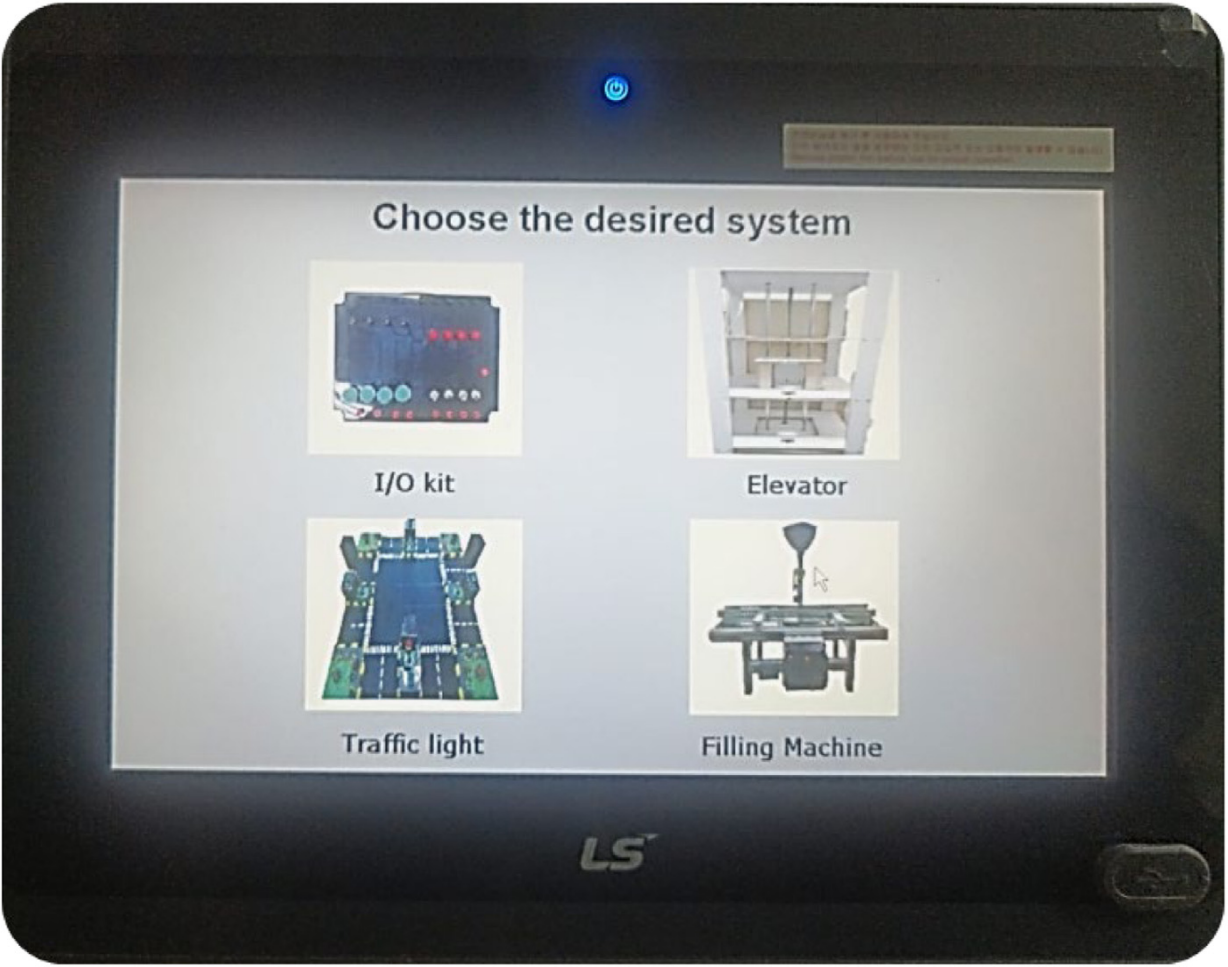
HMI displaying images of the primary kits—input/output, traffic light, elevator, and filling machine.

This configuration ensures an effective balance between resource efficiency and hands-on learning, enabling learners to gain practical experience with industry-relevant equipment while keeping laboratory expenses manageable. Detailed descriptions of the hardware components involved are provided in the following sections. The learners also have the flexibility to experiment with any of the application kits, which are preprogrammed into the HMI, as displayed in Fig. 2. These experiments are carefully designed to guide them through the necessary exercises and testing procedures. Once the learners develop a solution, which is verified by their instructor using one of the available application kits, they can move on to the next step: connecting the PLC to the selected kit. This hands-on phase allows learners to test and validate their simulated solution on actual PLC hardware, ensuring a smooth and effective transition from theoretical simulation to real-world application.

A key consideration in the design of the lab setup is the selection of components that balance cost, functionality, and ease of use. While open-source solutions such as OpenPLC offer significant cost advantages, several factors influenced our decision to use commercial PLC hardware instead:

• Ease of use for beginners: OpenPLC requires familiarity with software installation, configuration, and troubleshooting, which may pose challenges for students with limited programming or technical experience. In contrast, the selected commercial PLC provides a user-friendly interface and plug-and-play functionality, enabling students to focus on learning industrial automation concepts rather than debugging software issues.
• Hardware reliability and support: Commercial PLCs are typically backed by robust hardware warranties and technical support, ensuring reliability in educational settings where frequent handling by students may lead to wear and tear. OpenPLC, being software-based, relies on compatible hardware (e.g., Raspberry Pi or similar platforms), which may lack the durability required for repeated use in labs.
• Compatibility with existing educational resources: Many existing educational materials and curricula are designed around commercial PLCs, making it easier to integrate the proposed lab setup into established teaching frameworks. Using OpenPLC would require developing custom learning resources, which could increase development time and complexity.
• Real-world relevance: Commercial PLCs are widely used in industry, providing students with hands-on experience on equipment they are likely to encounter in professional environments. OpenPLC, while valuable for certain applications, does not replicate the exact user experience of industrial-grade PLCs.

While OpenPLC offers a cost-effective alternative, these considerations led us to prioritize commercial PLC hardware for this lab setup. Future work could explore hybrid approaches that combine the affordability of open-source solutions with the robustness and usability of commercial systems.

### Modular and application kit comparison

The proposed lab setup is built around two key components: The Modular Kit and the Application Kit, and the distinction between them is a foundation of the lab’s design. The Modular Kit serves as a reusable, standardized platform that includes a PLC and HMI, acting as the heart of the system where students can develop, simulate, and test their solutions in a controlled setting. This approach not only provides students with hands-on experience but also fosters collaboration and enhances problem-solving skills, as they work together to develop, simulate, and test their designs in a structured, iterative process. On the other hand, the Application Kits are specialized tools designed to mimic real-world industrial scenarios, like traffic light systems, elevators, or filling machines. These kits represent the practical side of learning, allowing students to take their simulated solutions and apply them to actual hardware. By sharing one set of Application Kits among all groups, we make the most of expensive equipment and avoid unnecessary redundancy. This clear separation between the two components creates a flexible and scalable system where new Application Kits can be added without needing to change the Modular Kits. Also, the distinction between the Modular Kit and Application Kit reflects a layered approach to educational automation: The Modular Kit focuses on the development phase, where students create and simulate solutions. The Application Kit focuses on the testing and validation phase, where students apply their solutions to real-world scenarios. Ultimately, this approach cuts costs, provides a well-rounded learning experience, and ensures the lab can grow and adapt to meet future educational needs, staying in line with the demands of modern engineering education.

### Design and rationale of the interface

The interface plays a pivotal role in the overall lab setup, acting as the critical bridge between the Modular Kit and the various Application Kits. It is intentionally designed to follow standardized and widely adopted industrial communication protocols, such as RS232 and RS485. This standardization ensures seamless interoperability, allowing any Modular Kit to connect effortlessly with any Application Kit, regardless of the specific experiment or hardware configuration.

Simplicity and usability are central to the interface’s design philosophy. Ports are clearly labelled, wiring is straightforward, and the software settings within the Human-Machine Interface (HMI) are intuitive and easy to navigate. This user-friendly approach minimizes setup time, enabling students to concentrate more on hands-on learning and experimentation rather than troubleshooting technical issues. Additionally, the interface is built for flexibility and scalability. Thanks to its plug-and-play architecture, new Application Kits can be introduced without modifying the Modular Kits. This modularity not only reduces initial and long-term hardware costs but also simplifies system maintenance and upgrades over time.

Beyond technical efficiency, the interface strongly supports the lab’s educational objectives. By incorporating industry-standard tools and practices, students gain valuable, real-world experience that enhances their readiness for careers in industrial automation. The adaptable design enables application across various scenarios, helping students apply their critical thinking and problem-solving skills in different industrial contexts.

### Modular-Kit design

The core kit of the proposed lab is a highly versatile and modular, purposefully engineered to support and enhance hands-on learning in industrial automation. While functional requirements shape the system's capabilities, several non-functional requirements have also been identified and utilized as the foundation for the design of the proposed kit, directly informing the development of key features and functionalities. to ensure the kit is well-suited for educational environments. These include the following considerations:

• Robustness: The kit is designed to endure frequent and sometimes rough handling by students during laboratory exercises. To ensure durability, the PLC is securely enclosed within a rugged plastic casing that protects it from physical damage during both transport and use. Furthermore, the kit utilizes banana plugs for wiring connections instead of traditional screw terminals, which are more susceptible to wear and mechanical failure over time. This design choice enhances reliability and extends the kit’s operational lifespan in an educational setting.
• Safety: The design prioritizes student safety by minimizing exposure to electrical hazards. All electrical components are securely housed within a protective plastic enclosure, significantly reducing the risk of accidental contact. Additionally, the use of banana plugs rather than exposed metal screw terminals further enhance safety by providing secure, insulated connections and minimizing the likelihood of short circuits or loose wiring during use.
• Ease of use: The kit is designed to streamline setup and operation, allowing students to concentrate on learning concepts rather than troubleshooting hardware issues. Its modular design enables easy transport and seamless integration of the PLC with any of the application kits, fostering a flexible learning environment. The use of banana plugs further simplifies the connection process, allowing for quick, secure, and tool-free wiring that does not require advanced technical skills, making it ideal for beginners and experienced users alike.
• Flexibility: The system is designed to accommodate a wide variety of experimental setups, enabling students to explore diverse industrial automation applications, such as traffic light control, elevator systems, and automated filling processes. This flexibility is made possible through a highly adaptable interface, featuring 12 inputs and 12 outputs connected via 24 banana plug terminals, along with two COM terminals that support both sinking and sourcing current configurations. This versatile I/O design allows seamless integration with a broad range of input and output devices, making the kit suitable for numerous practical exercises and real-world scenarios.

**Fig. 3:**
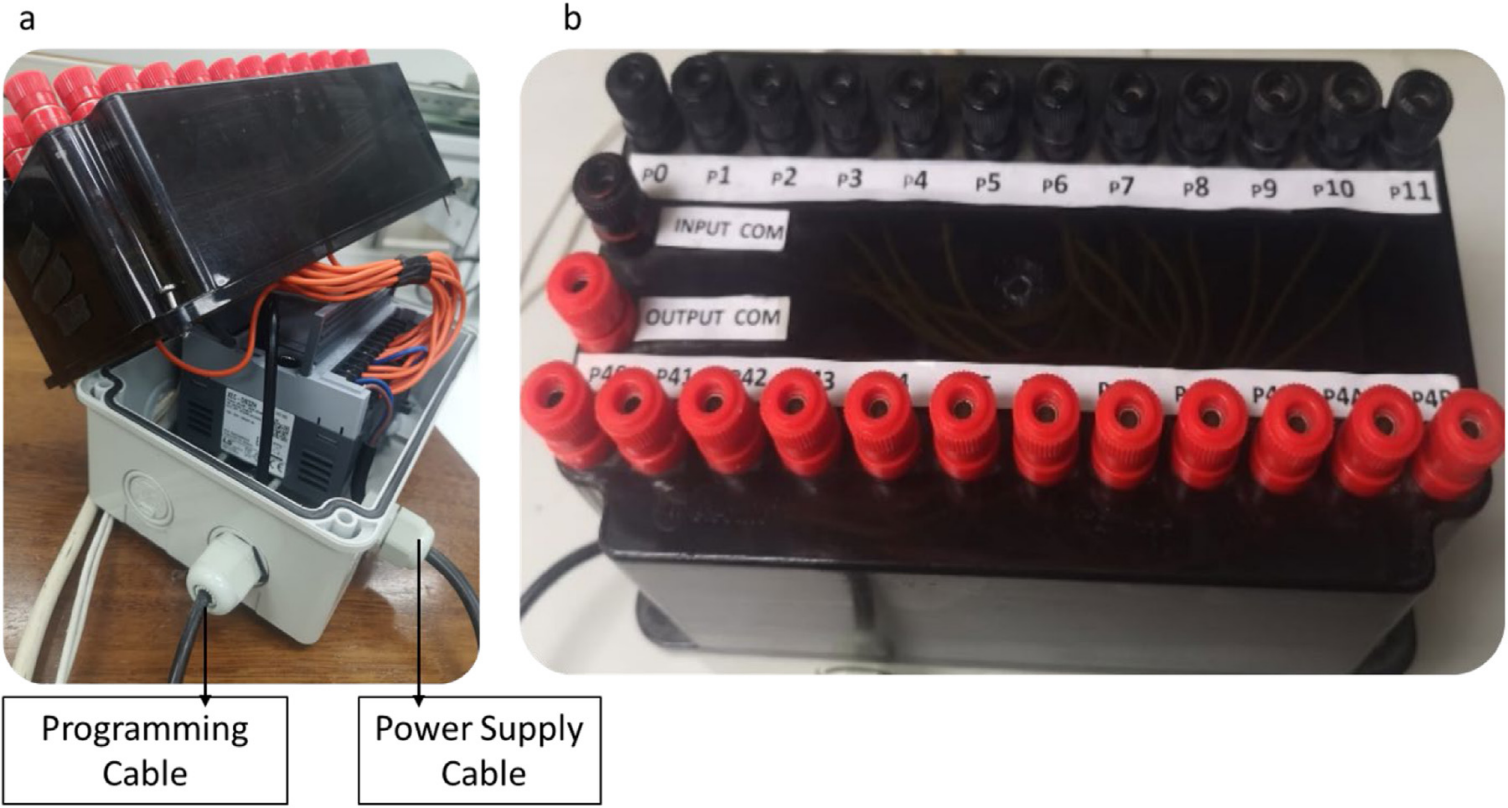
Top and side views of the modular PLC kit. a. side view, illustrating the power supply and programming cables; b. top view, showing possible connection terminals.

As shown in Fig. 3, the PLC is built around the XEC-DR32H PLC [17], which serves as the central component of the modular system. The kit is connected to external systems through two main cables: one for supplying 220 V AC power and another for programming. This setup ensures seamless integration with both power sources and software interfaces, offering a comprehensive platform for hands-on training in automation and control systems.

The Human-Machine Interface (HMI), provided by LG Extension from LS Electronics [18], serves as an essential complement to the PLC, offering an interactive platform for user-system communication. Its selection was based on a set of key criteria tailored to the needs of an educational Modular Kit:

• Ease of use: The HMI features an intuitive interface that enables students to interact with the system effectively, even with minimal prior training.
• Reliability: Built to endure the rigors of frequent classroom use, the HMI is robust enough to handle occasional mishandling or operational errors.
• Compatibility: It integrates seamlessly with the PLC, supporting real-time programming, control, and monitoring to ensure smooth and responsive operation.
• Cost-effectiveness: As part of a budget-conscious educational solution, the HMI offers excellent value without compromising on essential features or performance.

Together, these qualities make the HMI a practical and pedagogically effective component within the modular automation lab environment.

The selected HMI fulfils these requirements effectively, its user-friendly design includes clear visuals and straightforward navigation, enabling students to focus on learning rather than troubleshooting the interface. The robust construction ensures durability, even with frequent handling by students, reducing the risk of damage or malfunction. The HMI is specifically programmed to align with the PLC, ensuring seamless integration and real-time communication between the operator and the machine. Compared to other HMIs on the market, the LS Electronics HMI offers a cost-effective solution without compromising performance, making it well-suited for educational environments.

By integrating the aforementioned design requirements, the Modular Kit serves as a foundational educational tool for deepening learners’ understanding of programmable logic controllers (PLCs) and their role in industrial automation. It is specifically tailored to support introductory-level training, guiding students through the basics of PLC operation and programming within simplified, controlled environments. While the kit intentionally abstracts and simplifies real-world industrial scenarios, it lays a critical groundwork for learners to build upon. This foundation equips students with the essential knowledge and confidence needed to advance toward more complex tasks, including the configuration and management of PLCs in actual industrial settings.

### Application-Kits design

The Application Kits are designed to introduce trainees to foundational concepts and paradigms of industrial automation through practical, hands-on experimentation. Each kit focuses on a specific application—such as traffic light systems, elevators, and automated filling systems—and introduces key programming concepts that progressively build upon one another. For example, the traffic light system introduces basic ladder logic and sequential control, while the elevator simulation advances to more complex state-based programming. This structured progression ensures that trainees develop a comprehensive understanding of PLC programming principles and their real-world applications. In this section, the Input/output Practice Kit as well as three of the application kits are described, as follows:

#### Input/output practice kit

**Fig. 4:**
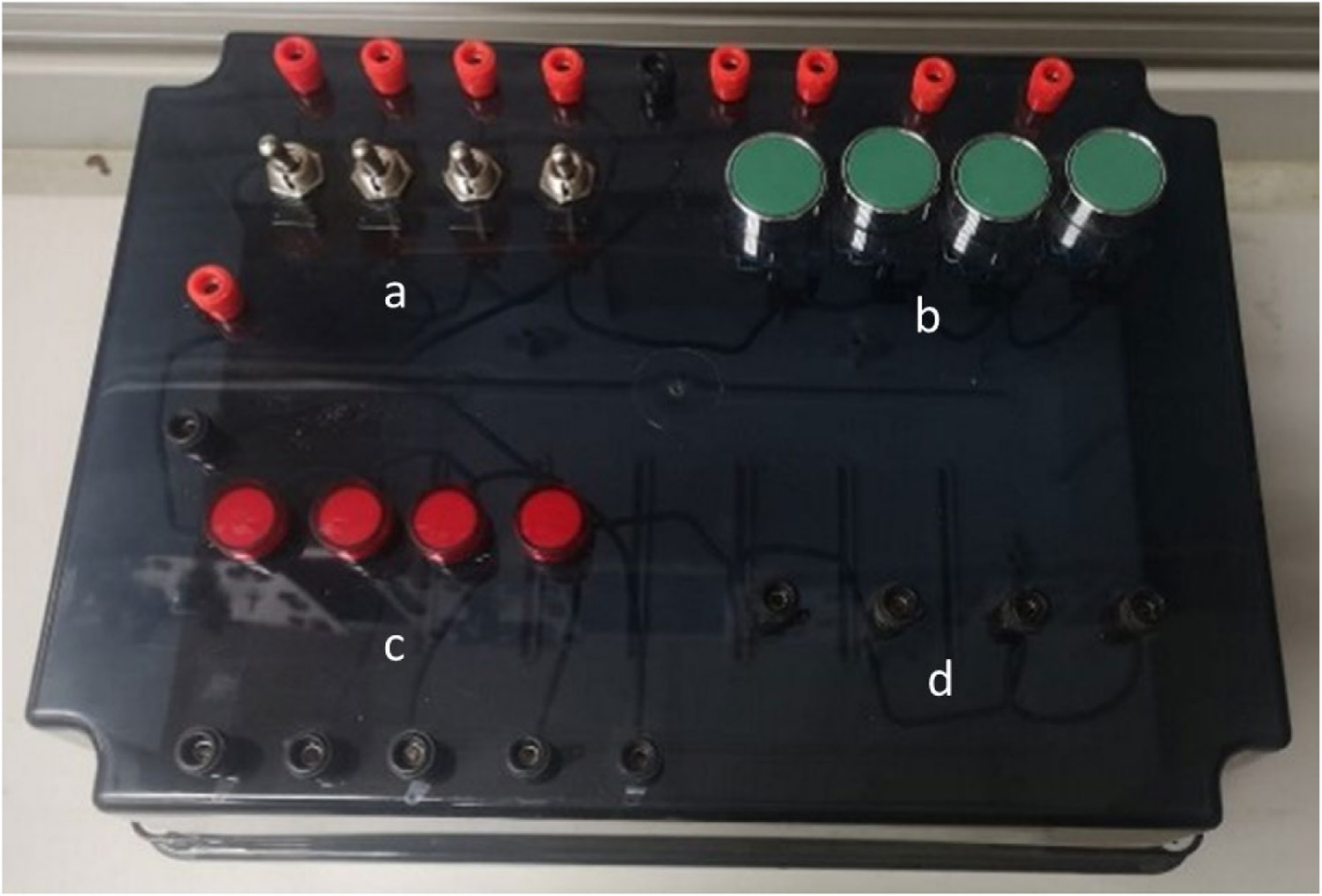
The Input/ Output practice kit, illustrating a. maintained switches; b. momentary pushbuttons; c. industrial LEDs; d. sockets.

The primary purpose of this interface is to familiarize learners with a variety of input and output configurations, including sinking and sourcing. It is also designed to help learners grasp fundamental bit instructions, such as ‘examine if closed,’ ‘examine if open,’ and ‘output energize.’ As shown in Fig. 4, the main box contains a range of inputs and outputs, offering a hands-on learning experience.

**Fig. 5:**
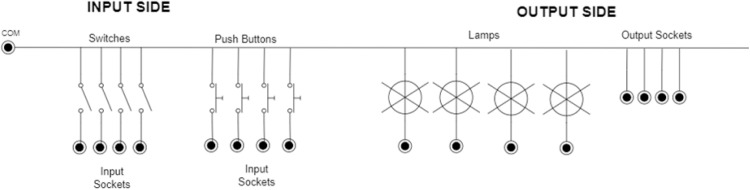
Sample wiring diagram of the input/output practice kit.

The input section includes both Mechanical Momentary inputs (four industrial pushbuttons) and Maintained inputs (four industrial switches). The output section features industrial lamps and sockets, allowing students to test and monitor their configurations. The output sockets are designed for versatility, enabling students to connect and control actuators or other devices beyond those embedded in the I/O box. The wiring diagram, shown in Fig. 5, illustrates the various inputs and outputs of this interface. Each component is connected to the COM terminal, which can either be 24 V or ground (GND), depending on whether the current configuration is sinking or sourcing. The other terminal is connected to the PLC. If the COM terminal is connected to 24 V and the PLC terminal is connected to ground, the PLC is configured for sinking. Conversely, if the COM terminal is connected to ground and the PLC terminal is connected to 24 V, the PLC is configured for sourcing.

#### Traffic-Lights kit

This kit is designed to control a four-way crossroad traffic lights system. It allows learners to explore and experience various traffic control scenarios while becoming familiar with both basic and advanced timer functions, as well as comparison instructions. The experiments that can be carried out on this kit make use of four industrial pushbuttons and twelve industrial lamps to simulate the traffic light signals. A custom wooden box is designed to host the various components of this application. The top face of the box serves as the platform to display the traffic lights and incorporate components such as the pedestrian pushbutton. The front face of the box serves as the user interface, featuring sockets for connecting to the PLC. For the traffic lights themselves, we created a 3D model using Fusion 360 and produced it through 3D printing.

**Fig. 6:**
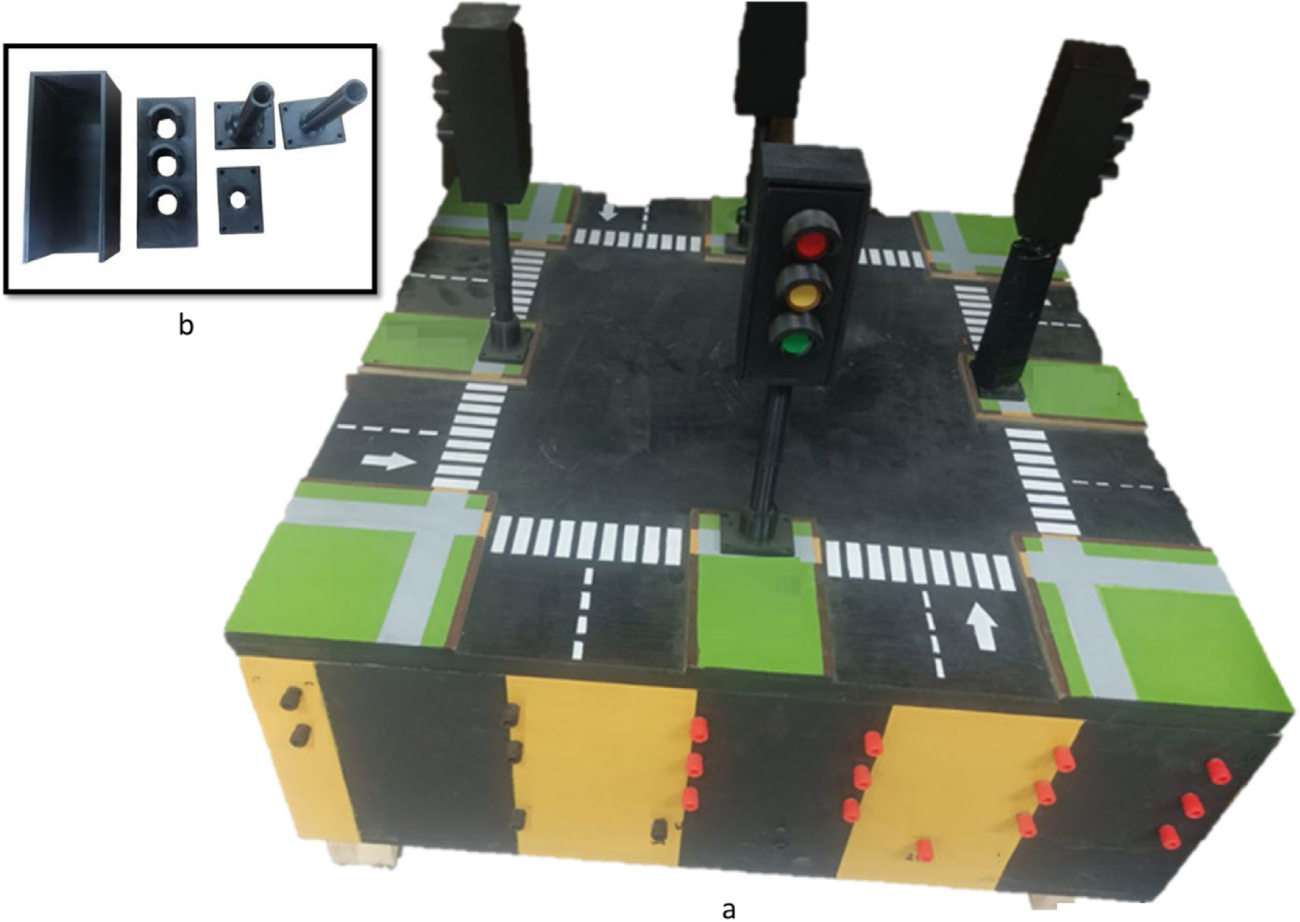
Traffic Light Kit, showing a. hardware design of the kit and in front the user interface with sockets for the traffic light model; b. 3D-printed components of a traffic light model.

**Fig. 7:**
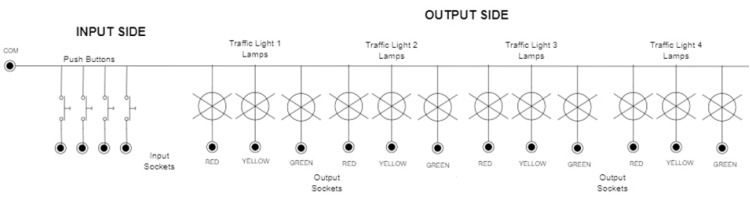
Example of a Traffic Light wiring diagram.

Fig. 6 displays the completed application, which offers a hands-on, interactive learning experience for students to apply their knowledge of automation systems in a practical setting. The wiring diagram for both the input and output sides of the traffic light system is shown in Fig. 7. Through this diagram, learners gain a deeper understanding of sinking and sourcing concepts by applying them in a real-world context.

#### Elevator kit

**Fig. 8:**
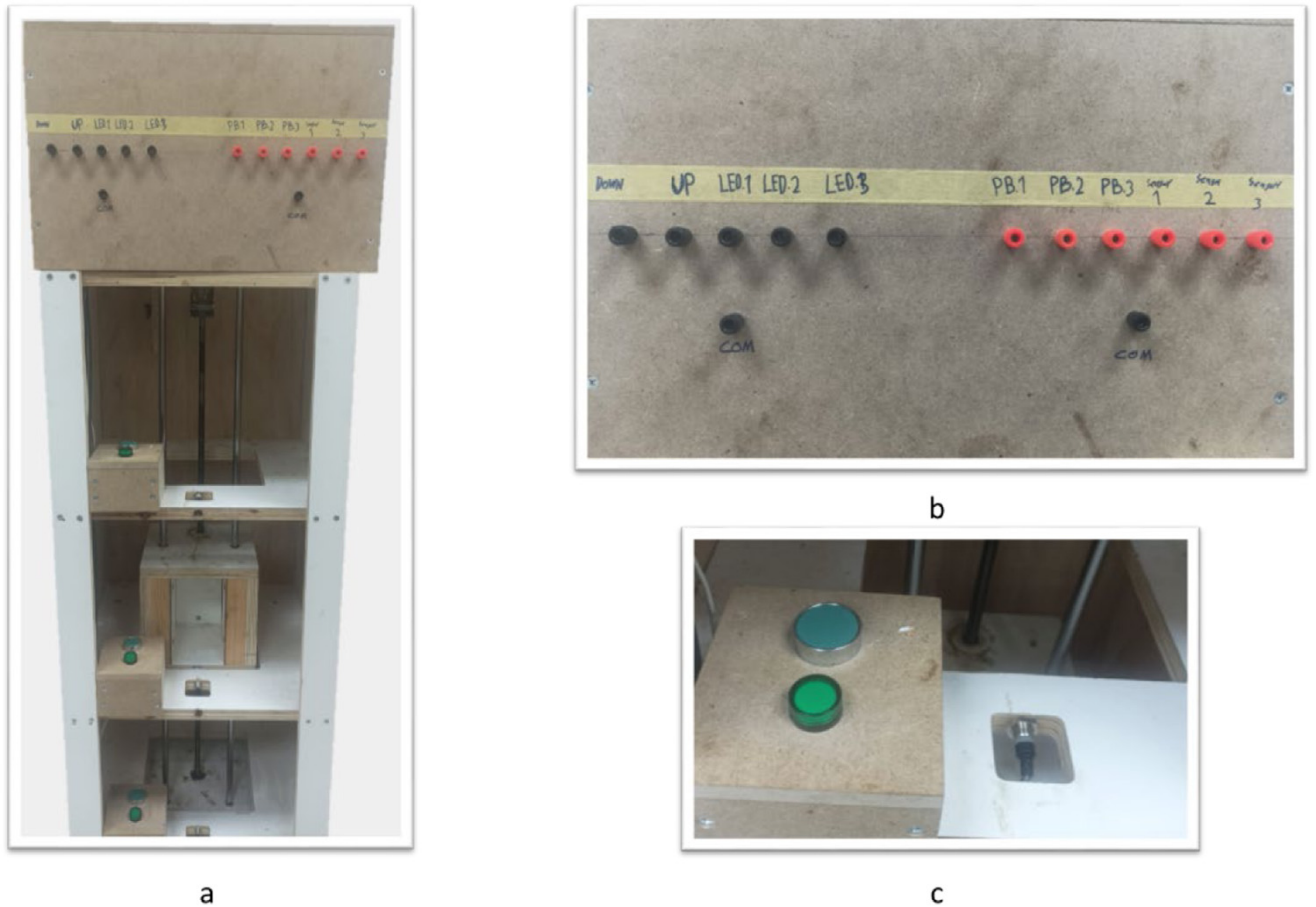
Elevator Kit, showing a. hardware design of the kit; b. user interface with socket connections; c. floor housings each equipped with a pushbutton, lamp and IR sensor to determine the cabinet position.

This kit, shown in Fig. 8, provides a more complex automation system compared to the previous applications. Learners will apply advanced programming techniques, utilizing instructions such as rising and falling edge detection and positive/negative transitioning sensing contacts. The outer structure of the kit is designed to replicate the rigidity of a real elevator body. It includes three industrial pushbuttons, three proximity sensors, three industrial lamps, and two contactors for motor control. The motor is connected to a power screw, which is attached to the elevator cabinet. This cabinet is supported by two metal rods, extending from the top of the final floor to the ground, ensuring stability during movement. The outer housing is constructed from wood, with a dedicated space for housing the motor and enclosing the wiring. The front face of the housing serves as the user interface, incorporating the necessary sockets for easy connectivity to the PLC. Each floor is equipped with a housing containing an industrial pushbutton and a lamp, allowing students to interact with and control the elevator system at different levels.

**Fig. 9:**
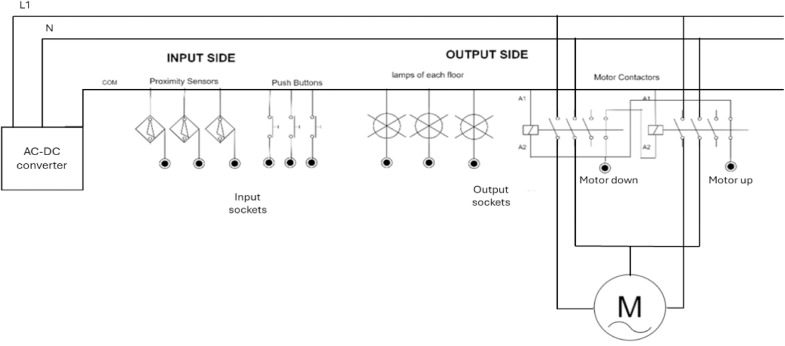
Sample wiring diagram of the elevator kit.

Fig. 9 illustrates the wiring diagram for the Elevator system. The input side includes three proximity sensors and three push buttons, allowing users to control and monitor the system. On the output side, the kit features a motor that drives the power screw and three lamps, one for each floor. Since the system includes an AC motor in addition to the DC components, we incorporated an AC-to-DC converter for compatibility. As the PLC cannot directly drive the AC motor, we used contactors to control the motor’s operation. To ensure the motor’s protection in the event of an error, we wired the contactors in a protection mode. Specifically, we connected the A2 terminal of each contactor to the normally closed switch of the other contactor, preventing both contactors from being activated simultaneously, which could potentially damage the system.

#### Filling machine kit

**Fig. 10:**
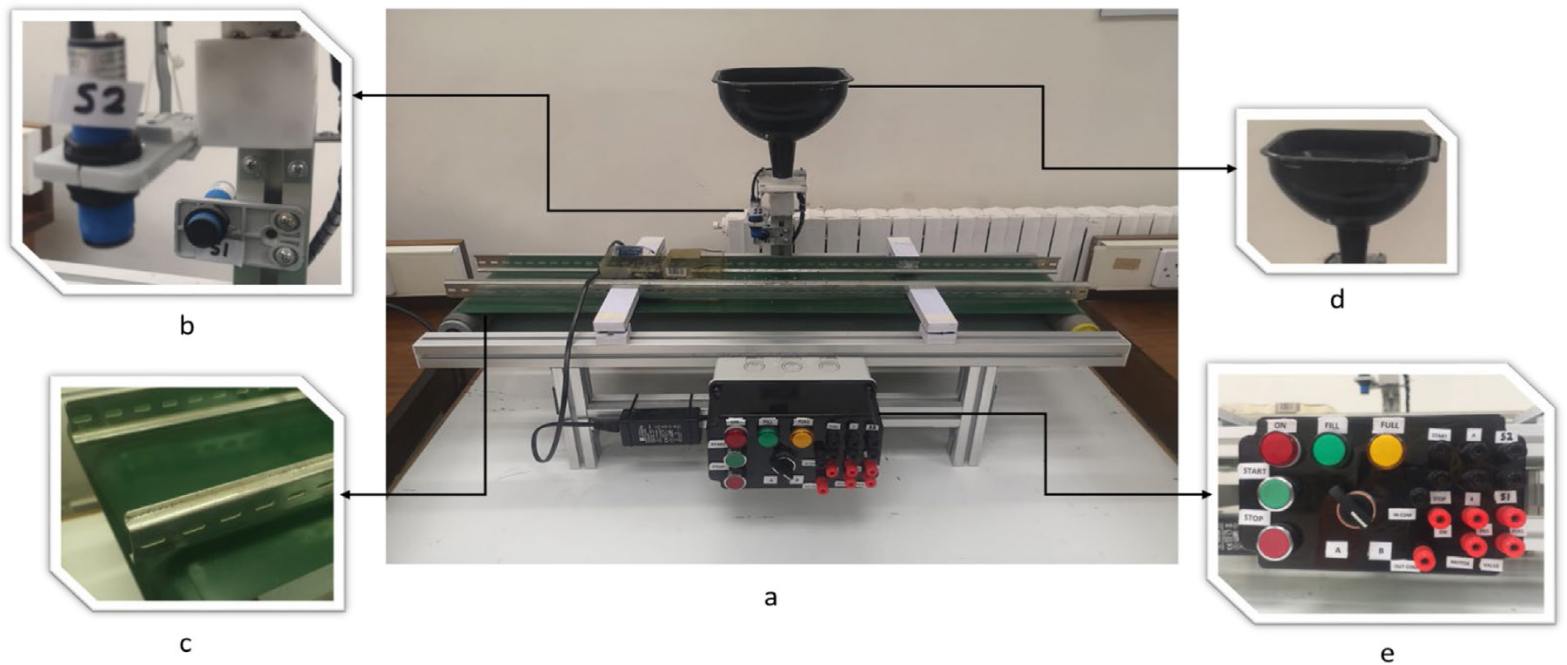
Filling machine kit, showing a. hardware design of the system; b. two diffused photoelectric sensors, c. PVC conveyor belt; d. funnel; e. a control panel.

This application allows learners to work with various types of counters and other control instructions. As shown in Fig. 10, the hardware setup consists of both mechanical and electrical components. The mechanical part includes a silo structure, a PVC conveyor belt, and a funnel. We selected aluminium profiles for the silo structure due to their resistance to rust and ease of repair, ensuring long-term durability. On the electrical side, the kit features a photoelectric sensor, actuators, and a control panel. The photoelectric sensor detects the presence of objects using light.

The filling machine is equipped with two types of diffused photoelectric sensors: One horizontally mounted to detect when the box arrives at a specific location to trigger the filling process, and another vertically mounted to monitor the material level inside the box. We incorporated a motor housed in a pulley system, referred to as an electric roller, along with an electric solenoid valve to control the material flow. After assembling the structure and integrating the sensors and actuators, we focused on managing the cables and wiring within the control panel, as shown in Fig. 10e. The control panel features pushbuttons, selector switches, LED lights, and connectors (banana plugs) to ensure a streamlined and efficient setup for operation.

**Fig. 11:**
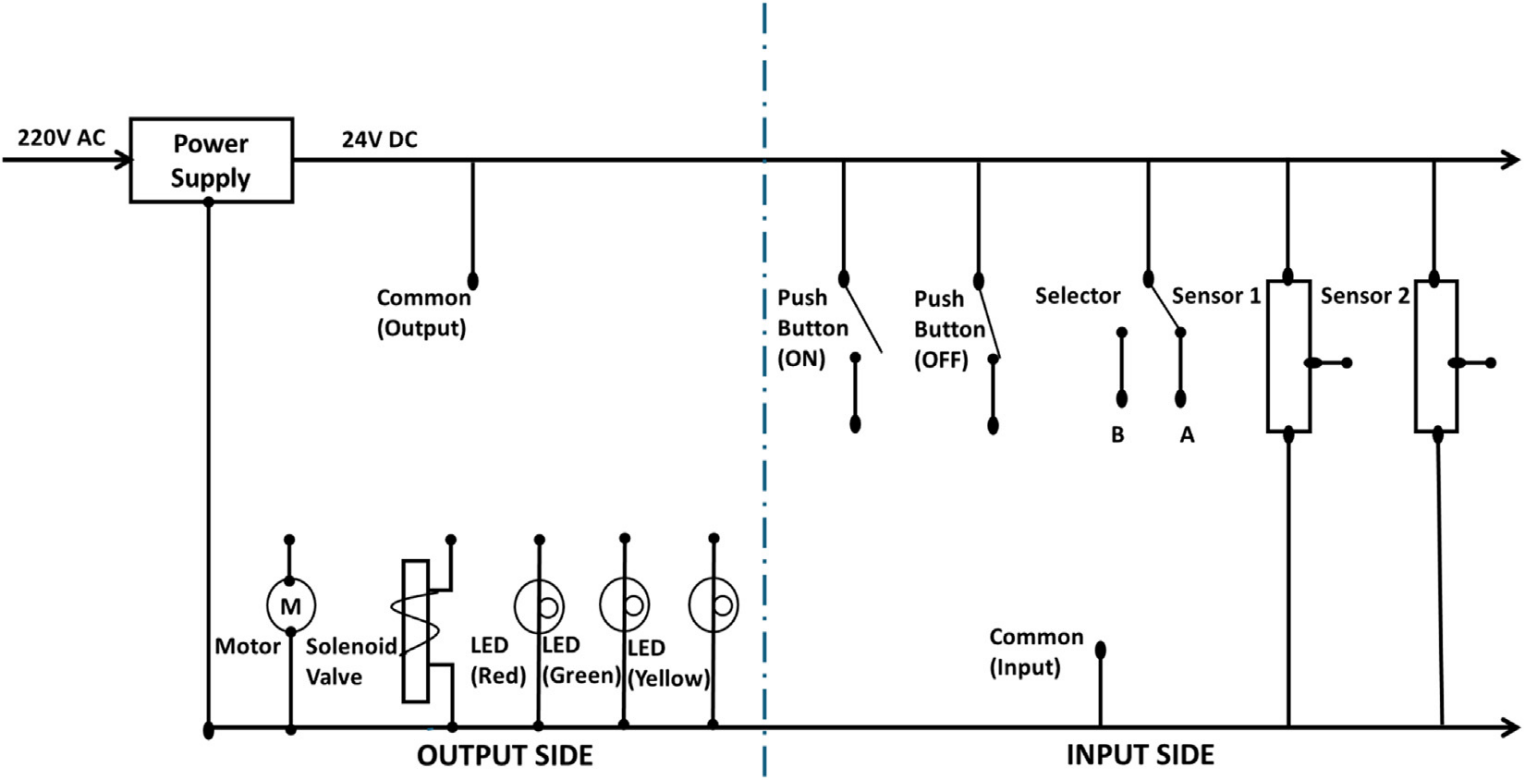
Sample wiring diagram of the filling machine kit.

All sensors, actuators, and input signals must be connected to a circuit powered by a reliable power supply. As shown in Fig. 11, the circuit is divided into two sections: The input side and the output side. The input side includes all devices that send signals to the PLC. These devices consist of two pushbuttons (start [NO] and stop [NC]), a selector switch with two signals (A and B), and two sensors. Each sensor operates at a rated voltage of 24 VDC and features three wires: one for the signal and two for power. The power wires are connected directly to the positive and negative terminals of the power supply, while the signal wire is connected to the corresponding input connector. The pushbuttons are wired to the positive terminal of the power supply, with a connector attached to the negative terminal. The negative terminal serves as the common ground for all input signals.

The output side consists of all devices that require a signal from the PLC to operate. All outputs are connected to a power supply capable of providing 24 VDC. These outputs include five loads: the motor, solenoid valve, and three LED lights. Each output is connected to the negative terminal of the power supply, while the positive terminal is connected to a common banana plug connector, which is shared by all the loads. To energize a specific load, the common connector is connected to the respective load, completing the circuit. This can be done either manually or through the PLC kit, which also includes connectors for seamless integration.

The application kits described in this work are primarily hardware-based, designed to provide hands-on experience with industrial automation concepts. While the kits themselves do not include proprietary software, any PLC programming code or testing scripts used for verification purposes will be made available upon request. Additionally, detailed documentation of the hardware design, wiring diagrams, and configuration instructions will be provided to ensure reproducibility and facilitate adoption by other educators or researchers.

## Method validation

This section presents experimental scenarios and test results of two developed kits, demonstrating their functionality, effectiveness and reliability. Each kit underwent rigorous testing to validate its design and ensure seamless operation. After finalizing the hardware design, we developed corresponding code for each kit using LS PLC's programming software, "XG5000” [19]. The programming was performed using ladder diagram language, a widely used format in industrial automation. The testing process involved verifying the seamless interaction between hardware and software components, including the PLCs, HMIs, sensors, and actuators. Each kit was subjected to rigorous testing under different operational conditions to ensure robustness and accuracy. The modular design allowed for efficient switching between experiments, further validating the practicality and adaptability of the lab setup.

To validate the functionality of the kits, testing was conducted to confirm proper wiring and operational performance. These tests confirmed that the kits are suitable for hands-on learning environments, ensuring that students can reliably use them for experimentation. While the kits are not inherently more robust than other setups, their modular design enhances flexibility and adaptability, allowing trainees to explore diverse scenarios without requiring specialized tools or extensive reconfiguration.

### Input/output practice kit

**Fig. 12:**
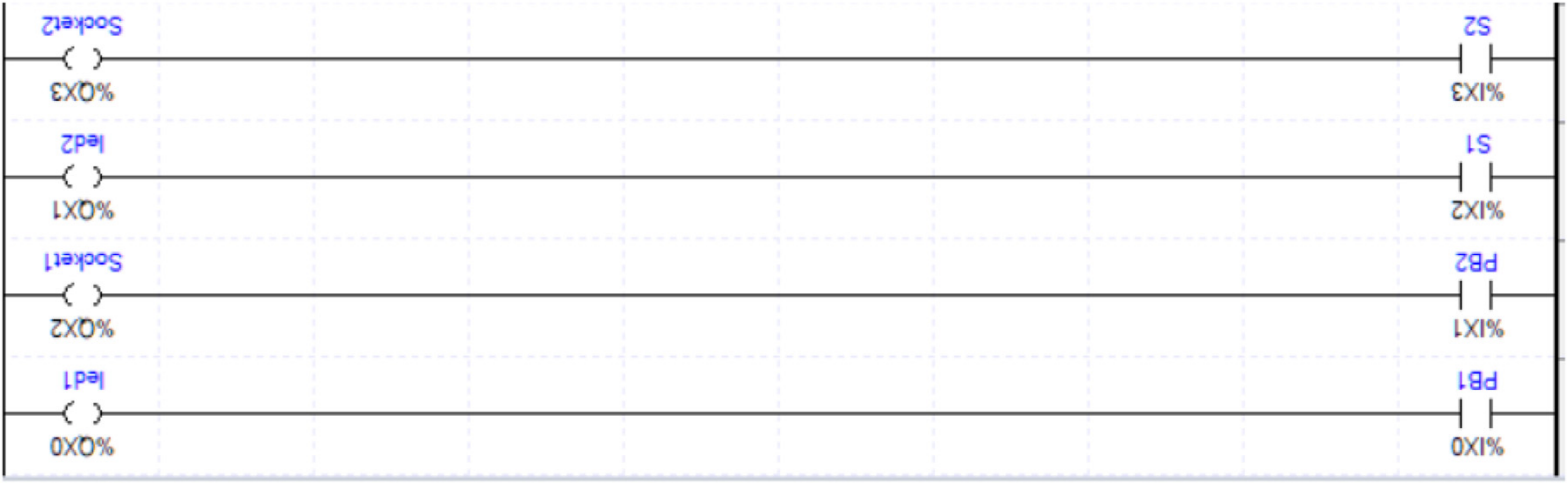
Code testing for the input/output kit.

In this experiment, a code was developed to test and validate the main components and wiring of the kit, ensuring that all inputs and outputs function as intended. The testing process focused on verifying the individual performance of each component, including push buttons, switches, LEDs, and output sockets. A sample ladder logic code, shown in Fig. 12, was created for this purpose using the “Examine if Closed” and “Output Energize” instructions, which are standard in PLC programming.

**Fig. 13:**
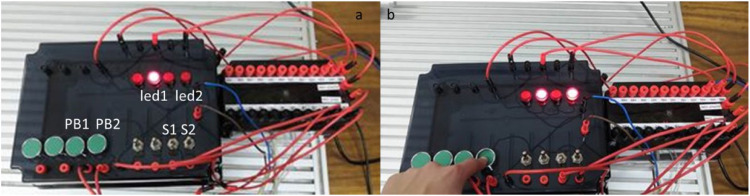
Testing setup for the input/output kit, showing a. switch test and b. push button test.

**Fig. 14:**
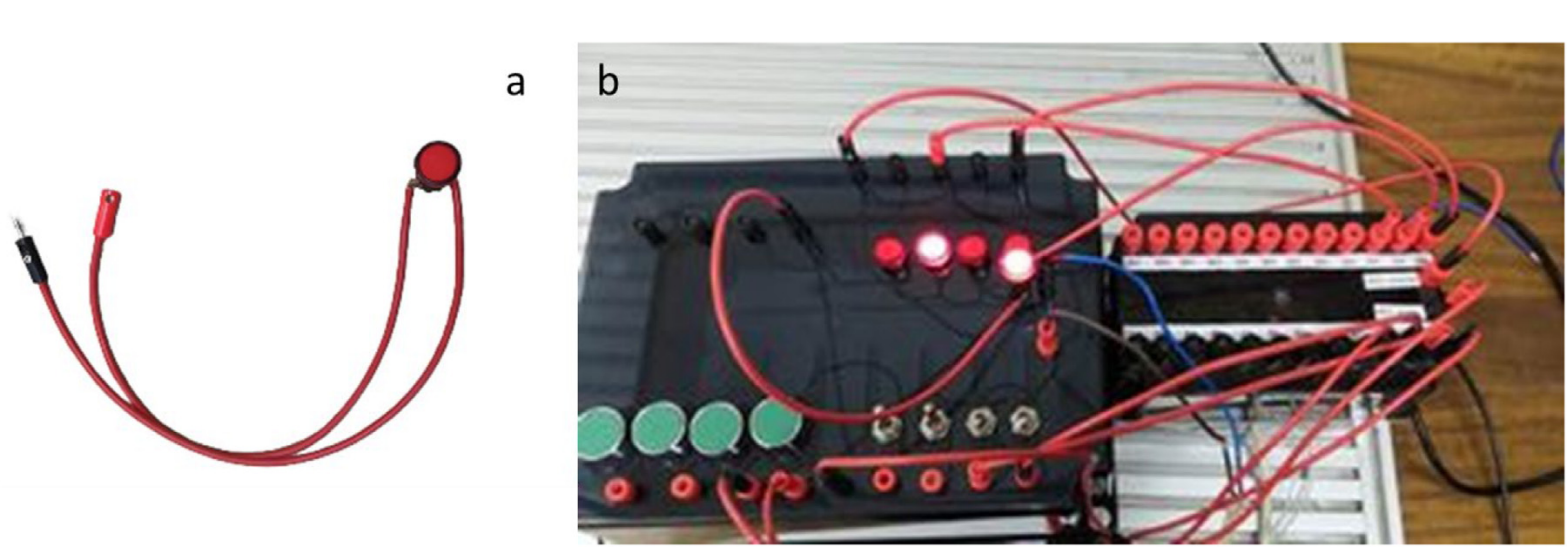
Wiring diagram, showing a. an output socket testing element and b. an output sockets testing setup.

The ladder logic was structured to test each input and output systematically. For example, when the push button PB1 is pressed, the led1 illuminates. However, because PB1 is a mechanical momentary button, LED1 turns off immediately after the button is released, demonstrating the behavior of a non-latching input. Conversely, the maintained switch S1 was programmed to activate led2 when switched on, and the light remains on until the switch is turned off, validating its latching functionality. The test setup is shown in Fig. 13, where a lamp was used as the output to visualize the results. Additionally, the PB2 and S2 inputs were tested by connecting them to the output sockets, which are designed to operate external components. For this part of the test, an external lamp was connected, with one terminal of the lamp attached to the output socket and the other terminal connected to the PLC. As depicted in Fig. 14, pressing PB2 or toggling S2 activated the external lamp, confirming that the output sockets could effectively power external devices.

These tests validated the correct wiring and functionality of the kit's components, demonstrating their reliability for hands-on learning. The ability to connect external components via the output sockets adds a layer of flexibility, allowing learners to experiment with additional devices beyond those integrated into the kit. This modular and adaptable approach enhances the educational value of the kit, providing learners with a comprehensive and interactive learning experience in automation concepts.

### Traffic lights kit

**Fig. 15:**
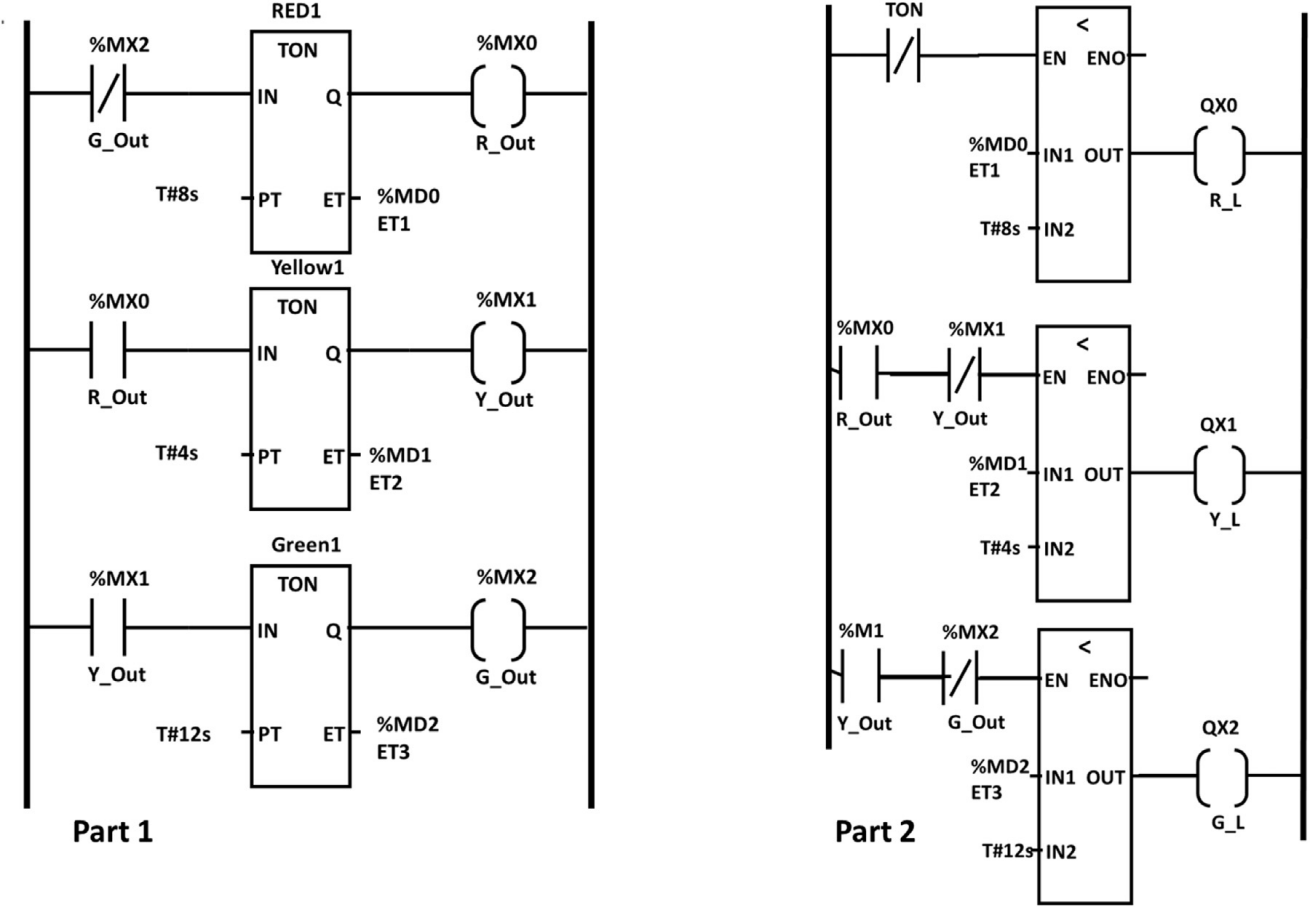
Testing code for single traffic light kit.

To test the functionality of the traffic light kit, we developed a ladder logic code to simulate the sequence of a single traffic light, ensuring proper timing and switching of signals. The code, illustrated in Fig. 15, utilizes timers and their associated control bits alongside “Examine if Open” and “Examine if Closed” instructions to control the operation of the traffic light system effectively. The code is divided into two main sections; timer configuration (Part 1) and logic for light activation (Part 2), which are outlined as follows.

(1) Timer configuration: Sets up three timers corresponding to the three traffic light signals: Red, Yellow, and Green, with durations of 12 s, 4 s, and 8 s, respectively. These timers are programmed to trigger sequentially, ensuring that each signal operates for its designated time. Upon completing its countdown, each timer activates its control bit, enabling a smooth transition to the next light in the sequence. This continuous cycle mimics the operation of real-world traffic control systems.
(2) Logic for light activation: Defines the activation conditions for each light based on the outputs of the timers. For example:
  • The red light activates when the yellow light’s timer cycle is complete
  • The yellow light is triggered at the conclusion of the green light’s timer
  • The green light switches on after the red light’s timer finishes its countdown

**Fig. 16:**
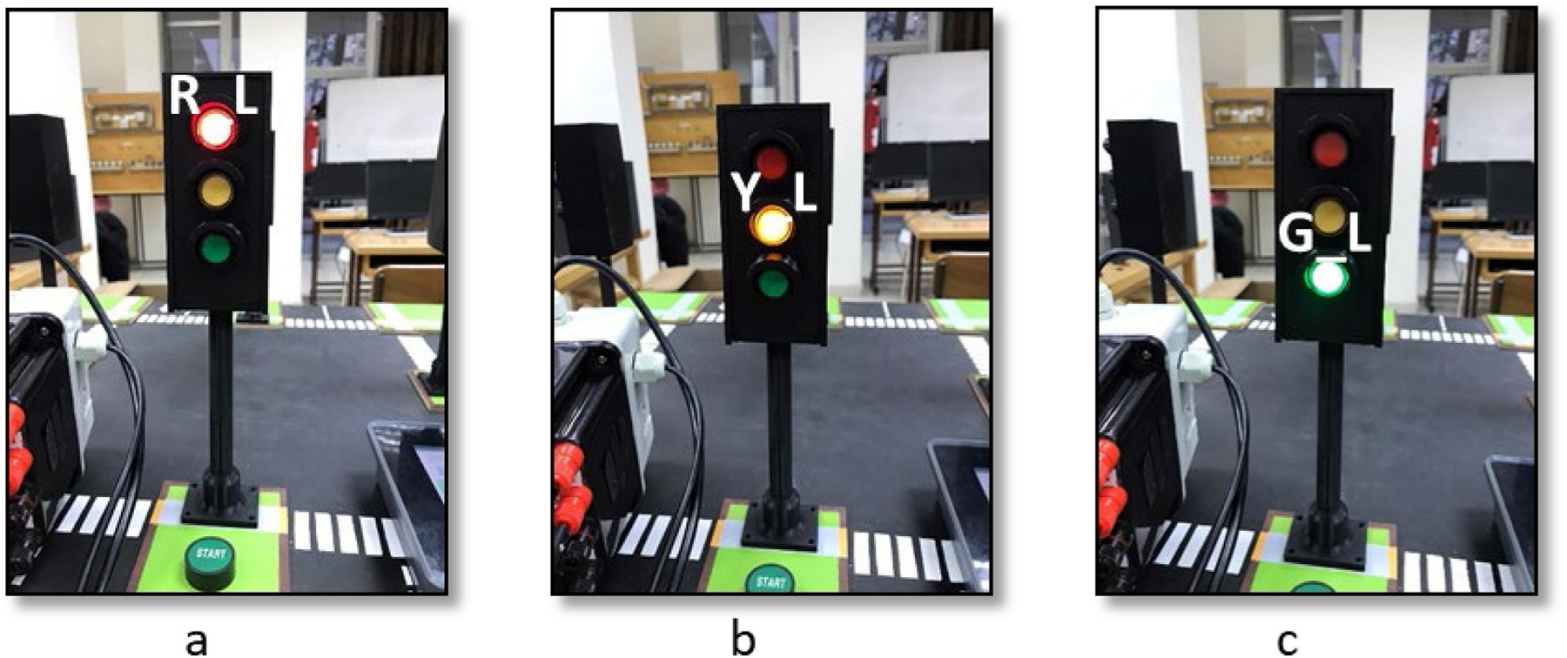
Traffic light sequence testing results, showing a. red, b. yellow, and c. green lights.

This carefully designed structure ensures that the lights transition in a logical and predictable sequence, effectively replicating the behavior of a real traffic light system while demonstrating fundamental automation principles. This kit was tested with the developed code to validate its performance. The results, depicted in Fig. 16, demonstrate the accurate timing and sequential operation of the red, yellow, and green lights. Each light turned on for its programmed duration, with precise transitions facilitated by the timers. This experiment showcases essential programming concepts such as timer functionality and condition-based control, which are fundamental in industrial automation.

### Cost evaluation of the proposed lab

To assess the cost-effectiveness of the developed hardware, we compared it with equivalent systems from various manufacturers in the market. The average prices of these commercial kits are provided in Table 1, based on data gathered by the authors from multiple manufacturers. This comparison reveals that the total cost of our proposed lab setup is approximately 27 % of the cost of traditional systems with similar features. This substantial cost reduction clearly demonstrates that the proposed solution offers a highly cost-effective alternative while maintaining the same functionality and educational value.

**Table 1:** Hardware cost comparison - proposed lab vs. existing systems.

Application Kit		The Proposed Solution			Existing Systems (Average)		
		Unit Price ($)*	Kits #	Cost ($)*	Unit Price ($)*	Kits #	Cost ($)*
Modular Kit	PLC	200	10	2000	n/a	n/a	n/a
	HMI	300	10	3000	n/a	n/a	n/a
I/O practice		50	1	50	150$	10	1500
Traffic Light		150	1	150	300$	10	3000
Elevator		350	1	350	750$	10	7500
Filling Machine		450	1	450	1000$	10	10,000
			Total	6000		Total	22,000

⁎ Average prices in this table are based on private communication with manufacturers.

### Analysis of cost drivers on traditional labs

Traditional educational automation labs often struggle with high costs due to three main issues: overcapacity of hardware, underutilization of resources, and a lack of modularity. In conventional setups, each group of students needs their own set of expensive equipment, such as different Application Kits. For instance, in a lab with 10 groups, this means purchasing 10 sets of Application Kits, which can cost around $22,000. Not only is this expensive, but it is also inefficient; many kits end up sitting idle when they are not being used by a particular group. On top of that, the absence of modularity makes it hard to share resources or scale up. Adding new experiments or accommodating more students requires buying even more hardware, driving costs even higher.

The proposed lab tackles these challenges head-on with a smarter, more cost-effective approach. Instead of providing each group with an individual Application Kit, we propose sharing a single set among all groups to significantly reduce redundancy and the associated cost. This simple change cuts down redundancy and reduces costs significantly. The key behind this efficiency is design modularity that relies upon a standardized interface to ensure compatibility across all kits. This allows seamless sharing and makes it easy to add new experiments without needing extra hardware. Students can work independently on their Modular Kits to develop solutions and then test them on the shared Application Kits, promoting collaboration and hands-on learning. As shown in Table 1, our solution reduces overall costs to just 27 % of what traditional systems require, while still delivering the same functionality and educational value. This approach offers a flexible, scalable, and affordable option for modern educational labs, addressing both budget constraints and practical needs.

## Concept-to-reality integration

The proposed lab is designed to bring together abstract ideas and real-world applications, ensuring that it is both theoretically solid and educationally impactful. At the heart of the system is modularity, where we use design principle that separates reusable Modular Kits from specialized Application Kits. This approach mirrors real-world engineering practices, where systems are broken down into independent, interchangeable components. By focusing on modularity, the lab achieves cost efficiency, scalability, and ease of maintenance, all while staying aligned with industry standards.

In practice, students work with Modular Kits equipped with PLCs and HMIs to develop and simulate their solutions. Once they’ve created a solution, they can test it on shared Application Kits that mimic real-world industrial scenarios, like traffic light control or elevator operation. This hands-on process helps students connect theoretical concepts to practical challenges, reinforcing their understanding and building valuable skills. Cost efficiency is another key feature of the system. Instead of providing each student group with its own set of expensive hardware, the lab centralizes the Application Kits. For instance, only one Traffic Light Control Kit or Elevator Kit is needed, no matter how many groups there are. However, as the number of groups increases, shared access may lead to queuing during testing phases, a factor that need be considered when implementing this approach in large-class settings.

This minimizes redundancy and keeps costs down without losing educational quality.

The system is also built to grow and adapt over time. New experiments can be added easily through plug-and-play compatibility. This means instructors can introduce new technologies or teaching objectives without needing to fix the entire setup. The flexibility ensures the lab remains future-proof and capable of keeping up with advancements in engineering education. Ultimately, by combining abstract concepts like modularity and scalability with practical, hands-on learning, the lab prepares students for real-world engineering challenges. Working with this modular, cost-efficient, and scalable system gives them experience in problem-solving, optimizing resources, and adapting to new situations—skills that are essential for careers in automation and engineering.

## Conclusion

This paper presented the development of a cost-effective and modular laboratory setup designed to provide students with practical, hands-on experience in industrial automation. The system enables multiple student groups to share a central core kit while utilizing modular components to simulate and test hardware experiments tailored to specific applications, including traffic light control, elevator systems, and automated filling processes. This approach effectively addresses the growing demand for affordable, flexible, and scalable training solutions within educational environments. The functionality of the developed kits is thoroughly evaluated and successfully validated for practical deployment in the automation laboratories at the University of Jordan School of Engineering. This validation involved a series of experimental scenarios designed to replicate real-world automation tasks. Each kit is rigorously tested to ensure correct operation, confirming its suitability for hands-on instruction and experiential learning.

Despite the notable flexibility and financial advantages offered by the developed solution, its impact on learning outcomes remains to be empirically verified. To address this, future work will involve conducting controlled experiments with students to evaluate the educational effectiveness of the developed kits. These experiments and others aim to establish clear metrics for assessing reliability, usability, and the overall impact on student learning, providing a data-driven foundation for broader implementation.

## Limitations

The current evaluation of the developed automation lab lacks long-term empirical data on learning outcomes, requiring future studies to validate its effectiveness across diverse student groups and curricula.

## Ethics statements

*‘Not applicable’.*

## CRediT author statement

**Musa Al-Yaman**: Conceptualization, Data curation, Formal analysis, Funding acquisition,Supervision and Writing - original draft. **Dana Alswaiti** and **Adham Alsharkawi**: Investigation, Methodology, Software and Visualization. **Majid Al-Taee**: Validation, Writing - review & editing.

## Declaration of competing interest

The authors declare that they have no known competing financial interests or personal relationships that could have appeared to influence the work reported in this paper.

## Data Availability

No data was used for the research described in the article.
